# Impact of Bacterial Siderophores on Iron Status and Ionome in Pea

**DOI:** 10.3389/fpls.2020.00730

**Published:** 2020-06-12

**Authors:** Tristan Lurthy, Cécile Cantat, Christian Jeudy, Philippe Declerck, Karine Gallardo, Catherine Barraud, Fanny Leroy, Alain Ourry, Philippe Lemanceau, Christophe Salon, Sylvie Mazurier

**Affiliations:** ^1^Agroécologie, AgroSup Dijon, INRAE, Université de Bourgogne, Université Bourgogne Franche-Comté, Dijon, France; ^2^RAGT 2n, Route d’Epincy, Louville-la-Chenard, France; ^3^Normandie Université, UNICAEN, PLATIN’, Esplanade de la Paix, Caen, France; ^4^Normandie Université, UNICAEN, INRAE, UMR 950 Ecophysiologie Végétale, Agronomie et Nutritions N, C, S, Esplanade de la Paix, Caen, France

**Keywords:** pea, *Pseudomonas*, siderophore, plant iron nutrition, pyoverdine, iron deficiency, IDC

## Abstract

Including more grain legumes in cropping systems is important for the development of agroecological practices and the diversification of protein sources for human and animal consumption. Grain legume yield and quality is impacted by abiotic stresses resulting from fluctuating availabilities in essential nutrients such as iron deficiency chlorosis (IDC). Promoting plant iron nutrition could mitigate IDC that currently impedes legume cultivation in calcareous soils, and increase the iron content of legume seeds and its bioavailability. There is growing evidence that plant microbiota contribute to plant iron nutrition and might account for variations in the sensitivity of pea cultivars to iron deficiency and *in fine* to seed nutritional quality. Pyoverdine (pvd) siderophores synthesized by pseudomonads have been shown to promote iron nutrition in various plant species (*Arabidopsis*, clover and grasses). This study aimed to investigate the impact of three distinct ferripyoverdines (Fe-pvds) on iron status and the ionome of two pea cultivars (cv.) differing in their tolerance to IDC, (cv. S) being susceptible and (cv. T) tolerant. One pvd came from a pseudomonad strain isolated from the rhizosphere of cv. T (pvd1T), one from cv. S (pvd2S), and the third from a reference strain C7R12 (pvdC7R12). The results indicated that Fe-pvds differently impacted pea iron status and ionome, and that this impact varied both according to the pvd and the cultivar. Plant iron concentration was more increased by Fe-pvds in cv. T than in cv. S. Iron allocation within the plant was impacted by Fe-pvds in cv. T. Furthermore, Fe-pvds had the greatest favorable impact on iron nutrition in the cultivar from which the producing strain originated. This study evidences the impact of bacterial siderophores on pea iron status and pea ionome composition, and shows that this impact varies with the siderophore and host-plant cultivar, thereby emphasizing the specificity of these plant-microorganisms interactions. Our results support the possible contribution of pyoverdine-producing pseudomonads to differences in tolerance to IDC between pea cultivars. Indeed, the tolerant cv. T, as compared to the susceptible cv. S, benefited from bacterial siderophores for its iron nutrition to a greater extent.

## Introduction

Iron (Fe) is a micronutrient essential for living organisms, microorganisms, plants and humans. It is indispensable to plant growth and crop iron content is a key determinant for human health (Briat et al., 2015). In soils, the total Fe often exceeds plant requirements but its bioavailability, being largely dependent on pH and redox conditions, is low in the circumneutral environments in which many crop plants are grown (Lemanceau et al., 2009). Iron bioavailability decreases sharply when pH increases with the result that iron is a limiting factor for plant growth in calcareous soils (i.e., as much as 30% of world soils) (Chen and Barak, 1982). Plants and microorganisms have therefore developed active strategies for iron uptake.

Microorganisms release siderophores that chelate iron and subsequently internalize the resulting complexes within their cytoplasm (Hider and Kong, 2010). Most research on iron nutrition in plants is focused on two active strategies: (i) strategy I found in non-graminaceous monocotyledons and dicotyledons and relying on rhizosphere acidification, reduction of Fe^3+^, and Fe^2+^ incorporation into the root, and (ii) strategy II found in grasses and relying on the excretion of phytosiderophores which scavenge Fe^3+^ before being incorporated into the root (Kobayashi and Nishizawa, 2012). However, this differentiation of plant species according to their iron uptake strategy is now known to be an oversimplification as both strategies contribute to iron nutrition in rice and peanut (Ishimaru et al., 2006; Xiong et al., 2013). In addition, fluorescent phenolic compounds produced by different plant species exert siderophore activity and represent a possible additional active strategy for both types of plant species especially in alkaline environments (Fourcroy et al., 2014; Schmidt et al., 2014).

Efficiency of plant iron nutrition varies considerably according to the plant crop (Hansen et al., 2006). For example, legumes (clover, soybean, chickpea, pea…) are known to differ greatly in their susceptibility to iron deficiency chlorosis (IDC) between species and between cultivars within a given species (Gildersleeve and Ocumpaugh, 1989; Zribi and Gharsalli, 2002; Mahmoudi et al., 2009; Helms et al., 2010). The variations in iron concentrations ([Fe]) of pea seeds, which range from 23 to 105 μg g^–1^ dry weight (DW) depending on the cultivar (White and Broadley, 2005), open up possibilities of Fe increase via breeding programs. Different plant traits have been proposed to account for this variability between legume cultivars. In soybean, genome wide association strategies (GWAS) identified quantitative trait loci (QTL) associated with IDC and underlined the multigenic character of this phenomenon. This approach also stressed the determinant influence of environmental factors on the plant ionome (Mamidi et al., 2011, 2014). In pea, DNA markers determining the mineral status of pea seeds have been identified and the effect of the plant’s field environment on the seed contents of Fe and other minerals has also been evidenced (Ma et al., 2017). Along with further progress in plant genetics and physiology, an increased understanding of these complex plant-environment interactions is therefore required.

Siderophore-mediated iron uptake by microorganisms has long been shown to contribute to plant nutrition under limiting conditions (Crowley et al., 1988; Crowley and Wang, 1991; Bar-Ness et al., 1992). More particularly Fe-pyoverdines, siderophores produced by fluorescent pseudomonads chelated to iron, are able to provide iron to strategy I and strategy II plants more efficiently than synthetic ferric chelate (Fe-EDTA) (Vansuyt et al., 2007; Jin et al., 2010; Shirley et al., 2011).

We therefore propose that further attention should be given to the contribution of the rhizosphere microbiota to plant iron nutrition. Indeed, protein families related to siderophore production were reported to be increased in root- and rhizosphere-associated bacterial taxa (Bulgarelli et al., 2015). More specifically, pseudomonad populations and their corresponding siderophores in the tobacco rhizosphere have been shown to differ according to iron nutrition of the plant genotype (wild type tobacco vs. mutant overexpressing ferritin) (Robin et al., 2006). These results showed that plants, closely related but distinguished by traits regarding iron nutrition, can differently impact specific members of the rhizosphere microbiota and consequently the pvds produced which in turn can differently impact the health of the host-plant (Robin et al., 2007). Model pyoverdines were shown to promote plant iron nutrition but very few of them have been tested for their impact on plant iron nutrition, even though pyoverdines are known to be highly diverse, with *c.a.*100 different structures described so far, and to differ in their activities (antagonism against fungal phytopathogens, induction of plant defense reactions) (Robin et al., 2007; van Loon et al., 2008; Cézard et al., 2015). Although there is a need for iron biofortification in grain legumes (Sperotto and Ricachenevsky, 2017), possible contribution of fluorescent pseudomonads and their siderophores to that biofortification needs to be explored. As regards pea iron status and ionome, the question arises as to whether the promotion of iron nutrition can vary with plant genotype and pvd type and whether the ionome can be modified since plant Fe status appears to influence the concentration of other elements (Cohen et al., 1998; Baxter et al., 2008; Maillard et al., 2016). We hypothesized that (i) distinct pvds could differently impact pea iron status and ionome and that (ii) different pea genotypes could be differently impacted by pvds. To test these hypotheses, we compared *in vitro* the effects of three distinct pvds on two pea cv. differing in their resistance to IDC (S, susceptible; T, tolerant) and the effect of inoculating a model strain producing one of the three pvds tested, and its pvd− mutant.

## Materials and Methods

### Microbial Strains and Culture Conditions

*Pseudomonas fluorescens* C7R12 (Eparvier et al., 1991; Lemanceau and Alabouvette, 1991) is a model strain already used to investigate the effects of its siderophore pyoverdine (pvdC7R12) on plant iron nutrition (Vansuyt et al., 2007; Shirley et al., 2011; Trapet et al., 2016). Thus pyoverdine contribution to bacterial competitiveness and plant iron nutrition (i.e., *Arabidopsis*, tobacco, fescue rye grass wheat and barley) was previously demonstrated using a pyoverdine-minus (pvd−) mutant PL1 of C7R12 (Mirleau et al., 2001; Landa et al., 2002; Trapet et al., 2016). *Pseudomonas* strains D4214 and B426 isolated from the rhizosphere of Dexter and Balltrap, respectively, were selected since their pyoverdines (as identified by isoelectric focusing according to Meyer et al., 2008, data not shown) were representative of those of *Pseudomonas* from the rhizosphere of Dexter and Balltrap, respectively. *Pseudomonas* strains were grown routinely on King’s medium B (KBM) (King et al., 1954) or in KBM broth at 25°C. For pyoverdine extraction bacteria were grown in succinate liquid medium (Meyer and Abdallah, 1978) at 25°C with shaking at 200 rpm for 72 h.

### Pyoverdine Purification and Ferri-Pyoverdine Preparation

Pyoverdines pvdC7R12 from *P. fluorescens* C7R12, pvd2S from *Pseudomonas* sp. D4214, and pvd1T from *Pseudomonas* sp. B426 were obtained from cultures grown in succinate medium (Meyer and Abdallah, 1978) at 25°C with shaking at 200 rpm for 72 h then extracted from bacterial supernatants following the purification protocol previously described by Hartney et al. (2013). Briefly, the supernatants were passed through a column of Amberlite XAD-4 before elution with 100% methanol and drying. A second chromatography step was carried out in a column of LiChroprep RP-18 rinsed with EDTA and then acidified water (pH 4.0) before elution of the pyoverdines with 80% methanol followed by their concentration and freeze-drying prior to storage at 4°C in the dark. Ferripyoverdines (Fe-pyoverdine or Fe-pvd) were obtained by mixing the purified pyoverdines with inorganic FeCl_3_ at a molar ratio of 1:1. The amount of pvd required to chelate iron in a solution 1:1 has been determined experimentally. Based on the fact that pvds chelate iron in equimolar amounts (Meyer and Abdallah, 1978), increasing concentrations of FeCl_3_ were added to a solution of apo-pvd prepared in acetate buffer (pH 5.2). Absorption spectra were recorded between 320 and 480 nm to identify the concentration of iron necessary to obtain the disappearance of the apo-pvd peak (c.a. 380 nm) and the maximum absorption peak for Fe-pvd (c.a. 410 nm). Concentrated Fe-pyoverdine solutions (600 μM) were prepared, filtered, sterilized and stored at 4°C in the dark.

### Plant Growth Conditions and Sampling

Two cultivars of *Pisum sativum*, Dexter and Balltrap, were chosen for their contrasting tolerance to iron deficiency chlorosis (IDC) in field conditions: Balltrap being tolerant and Dexter susceptible (P. Declerck, personal communication) as confirmed in a large-scale cohort study which compared field and greenhouse results (PersPEAcase RAGT-INRAE projet P. Declerck, C. Jeudy & C. Salon; personal communication). These two cultivars are hereafter indicated T for tolerant (Balltrap) and S for susceptible (Dexter).

#### Field Experiment

A field experiment was performed in a calcareous loam soil (Montardoise, France) with a low extractible iron content (DTPA-Fe = 10.3 ± 0.34 mg kg^–1^) and a high pH (8.27 ± 0.08 in water) (main soil characteristics are presented in Supplementary Table S1) in order to (i) compare both pea cultivars selected for their susceptibility to IDC and growth in conditions of low iron bioavailability, (ii) isolate strains of pseudomonads from the roots of the two cultivars, and (iii) produce seeds of both peas in similar conditions to allow their comparison during *in vitro* bioassays. For each cultivar, four plots 1 × 5 m in size were sown at the beginning of October 2016 (120 plants per m^–2^). Visual chlorosis scores ranging from 1 to 9 (1, no yellowing; 3, mild yellowing; 5, moderate chlorosis; 7, severe chlorosis; and 9, severe chlorosis and necrosis) were recorded three times per plot after 22 weeks and 24 (flowering time) weeks. At weeks 24 (flowering time) and 33 (harvest time), five plants were randomly sampled per plot and pooled. The shoots and roots of these samples were separated. Roots were washed twice in 100 ml of sterile milliQ water and all nodules were removed. Shoots and roots were oven-dried separately at 60°C to constant weight (dry weight, DW). At harvest time, the seeds yielded by the samples were collected and dried separately.

#### *In vitro* Bioassays

*In vitro* bioassays were conducted in order to differentiate the responses of S and T cultivars to ferripyoverdine supplementation and bacterial inoculation (WT strain and pvd− mutant). After surface sterilization by gentle shaking in 70% ethanol for 5 min and in calcium hypochlorite (1%) for 15 min, the seeds were then rinsed successively three times for 5 min in sterile demineralized water. They were then soaked in sterile demineralized water for 2 h and germinated in the dark at room temperature in sterile Petri dishes containing ash-less sterilized filter paper sheets impregnated with demineralized water. One germinated seed was put on an agar slant tube loosely capped with gauze-wrapped non-absorbent cotton wool (day 1) which allowed the shoots to grow outward. The agar slant tubes with germinated seeds were covered with opaque black plastic to protect the roots from light and placed in a growth chamber under a 16 h photoperiod (300 μmol m^–1^ s^–1^; 23°C/20°C). The agar slant tube (diameter, 2 cm; height, 15 cm) contained 30 mL of Hoagland medium (pH = 6) (5 mM KH_2_PO_4_, and Ca(NO_3_)_2_, 2,5 mM KNO_3,_ 1 mM MgSO_4_, 50 μM H_3_BO_3_, 5 μM MnSO_4_, 15 μM ZnSO_4_ 7 H_2_O, 3 μM Na_2_MoO_4_ 2 H_2_O, 2.5 μM KI(H_2_O)_7_, and CuSO_4_ 5 H_2_O) solidified with 15 g L^–1^ of agar (Sigma A1296 agar, Sigma-Aldrich, St. Quentin Fallavier, France). The residual iron in the agar medium was 12.52 ± 0.84 μM (corresponding to 0.7 ± 0.05 mg kg^–1^) as measured on three dried samples (see below for method).

In the Fe-pyoverdine supplementation bioassays, on day 7, plants were supplemented with 8 mL of diluted (1/4) Hoagland’s solution with or without Fe. The treatments were as follows: 0 Fe (non-supplemented), Fe-EDTA (15 μM), Fe-pvdC7R12 (15 μM), Fe-pvd1T (15 μM), and Fe-pvd2S (15 μM). Six plants were harvested per treatment on day 24 and pooled in pairs.

In the bacterial inoculation bioassays Hoagland’s agar was supplemented with Fe-EDTA (1 μM). On day 1, plants were (i) inoculated with C7R12 (WT pvd+) or with PL1 its mutant pvd− (10^7^ colony-forming units, CFU, per tube) or (ii) non-inoculated, i.e., supplemented with a volume of sterile water equivalent to that of the inoculants. Nine plants were harvested per treatment and pooled in threes.

The shoots and roots of plants grown in the bioassays were oven dried separately at 60°C to constant weight.

### Determination of the Ionomic Composition of Pea Plants

Dry samples were ground into fine powder using a Retsch mixer mill mm400 homogenizer (Retsch, Eragny, France). The concentrations of 13 elements (B, Na, Mg, P, S, K, Ca, Mn, Fe, Co, Cu, Zn, and Mo) were measured by High Resolution Inductively Coupled Plasma Mass Spectrometry (HR ICP-MS, Thermo Scientific, Element 2^TM^, Bremen, Germany) following microwave acid sample digestion as previously described by Maillard et al. (2016) and modified as follows. All samples were spiked with two internal-standard solutions of gallium and rhodium for final concentrations of 5 and 1 μg l^–1^, respectively, diluted to 50 ml with Milli-Q water to obtain solutions containing 2.0% (v/v) of nitric acid, and then filtered at 0.45 μm using a teflon filtration system (Digifiltre, SCP Science, Villebon-sur-Yvette, France).

### Extraction, Separation and Relative Quantification of Seed Proteins

Total soluble proteins were extracted from four seed samples, each consisting of 30 dry mature seeds per experimental treatment, using 500 μl of the urea/thiourea buffer for 10 mg of seed powder, as previously described (Gallardo et al., 2007). For each seed sample, 10 μg proteins were separated by one-dimensional electrophoresis (1-DE) in a 12% sodium-dodecyl sulfate polyacrylamide gel using the XCell4 *Surelock*^TM^ Midi-Cell system (Life Technology, Illkirch, France) and stained with Coomassie Blue R250. Image acquisition was performed using the Odyssey Infrared Image System scanner (LICOR Biosciences, Lincoln, NE, United States) with an intensity of 7.5 and a resolution of 169 μm. Protein band detection and quantification were performed using Phoretix 1D (v11.2, TotalLab Limited, Newcastel, United Kingdom). The quantitative data for each well were normalized by dividing the volume of each protein band by the total band volume. The molecular weight, in kilodaltons (kDa), of each protein band was calculated using the pre-stained low-range protein ladder (Bio-Rad, Marnes-la-Coquette, France). Protein annotation was performed by comparison with previously established 1-DE maps of pea seed proteins (Henriet et al., 2019).

### Data Analyses

One-way ANOVA was used for all comparisons (i.e., roots, shoots, whole plants and seed biomasses; visual IDC scores; iron concentration; concentrations of other elements; root:shoot ratio of iron concentration, [Fe] R:S ratio; protein band volume to total protein volume; percentages of increase in iron concentration). ANOVA assumptions were verified through Shapiro-Wilks test for normality of distributions and Bartlett’s test for homogeneity of variance. When ANOVA assumptions were not met, a Kruskall-Wallis test was performed as indicated in the results presentation. The [Fe] R:S ratio and increase (%) of iron concentration were subjected to arsine transformation prior to statistical analysis. The percentage increase of iron concentration after supplementation with Fe-EDTA or Fe-pyoverdine (pvdC7R12, pvd1T, pvd2S) was calculated using the formula: [([Fe] of supplemented sample - [Fe] of corresponding non-supplemented control): ([Fe] of corresponding non-supplemented control)] × 100; where [Fe] is the iron concentration expressed in g kg^–1^. A two-way ANOVA was applied in bacterial inoculation bioassays to assess the significance of the effects of the cultivar and of bacterial inoculation on the biomass and iron concentration of roots, shoots and the whole plant grown *in vitro*. Data were analyzed using R studio version 1.1.456 software and the stats (3.4.2) package.

## Results

### Pea Growth, Seed Yield, Iron Concentration, Ionome in S and T Cultivars Cultivated Under Field Conditions

Although shoot mass (per plant DW: S, 4.14 ± 0.49 g and T, 4.45 ± 0.40 g; *F* = 1.031, *p* = 0.349) and seed yield (S, 7.4 ± 0.85 t ha^–1^ and T, 7.6 ± 1.17 t ha^–1^; *F* = 0.168, *p* = 0.696) were higher in T than in S at harvest, these differences were not significant. Chlorosis symptoms, as indicated by the visual IDC score, were significantly higher in S than in T at the two recording dates, i.e., week 22 (S, 4.4 ± 0.64 and T, 1.2 ± 0.15; *F* = 80.77, *p* = 0.003) and during the flowering period (week 24) (S, 2.2 ± 0 and T, 1.2 ± 0.15; *F* = 82.371, *p* = 0.003).

**FIGURE 1:**
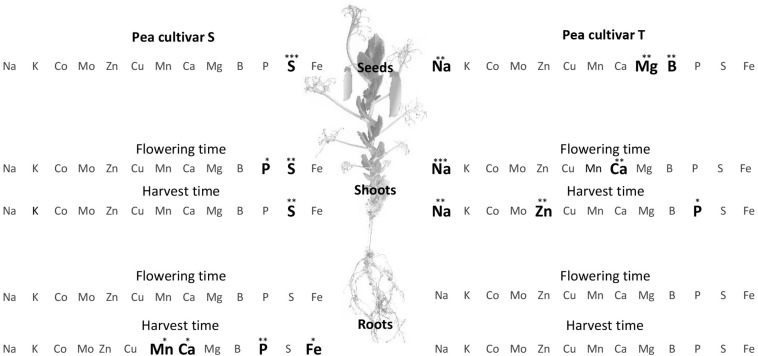
Comparison of elemental concentration in two pea cultivars, tolerant (T) and susceptible (S) to iron chlorosis, grown in a calcareous loam soil under field conditions (*n* = 4). Bold characters highlight those elements for which the concentrations were significantly higher in one cultivar, as compared to the other, within a same plant compartment and at a same sampling date. ANOVA *p*-value: *p < 0.05; **p < 0.01; ***p < 0.001. Data regarding the element compositions of mature seeds are available in Supplementary Figure S1A.

At harvest, the iron concentration was significantly higher in S roots (S, 541.24 ± 83.11 μg g^–1^ DW and T roots, 422.95 ± 34.92 μg g^–1^ DW; *F* = 6.888, *p* = 0.039), leading to a significantly higher root:shoot iron concentration ratio in S than in T ([Fe] R:S ratio). The S and T cultivars also differed in their concentrations of other ions (Figure 1). Root manganese ([Mn]), calcium ([Ca]), and phosphorus ([P]) concentrations were significantly higher in S than in T at harvest time (Figure 1), and the shoot and seed sulfur concentrations ([S]) were higher in S than in T at harvest and also at flowering time. In contrast, the shoot sodium concentration ([Na]) was significantly higher in T, at both sampling dates, and the seed magnesium ([Mg]) and boron ([B]) concentrations significantly higher in T at harvest time (Figure 1).

The total soluble protein content of mature seeds did not differ between the two cultivars (S, 32% ± 0.05 and T, 33% ± 0.04; *F* = 0.034, *p* = 0.861), and their protein compositions varied only slightly, with a higher relative abundance of two proteins annotated as vicilins (7S globulins) (+21% for each) in S, and of an unidentified protein (+16%) in T (Supplementary Figure S1). Seeds of cultivars S and T were harvested for the experiments presented above and their iron contents were shown to not differ significantly (S, 9.45 ± 1.86 μg per seed and T, 10.80 ± 1.55 μg per seed; *F* = 1.241, *p* = 0.307).

### Impact of Ferrisiderophores on Pea Growth, Development, Iron Content and Ionome in S and T Cultivars

**FIGURE 2:**
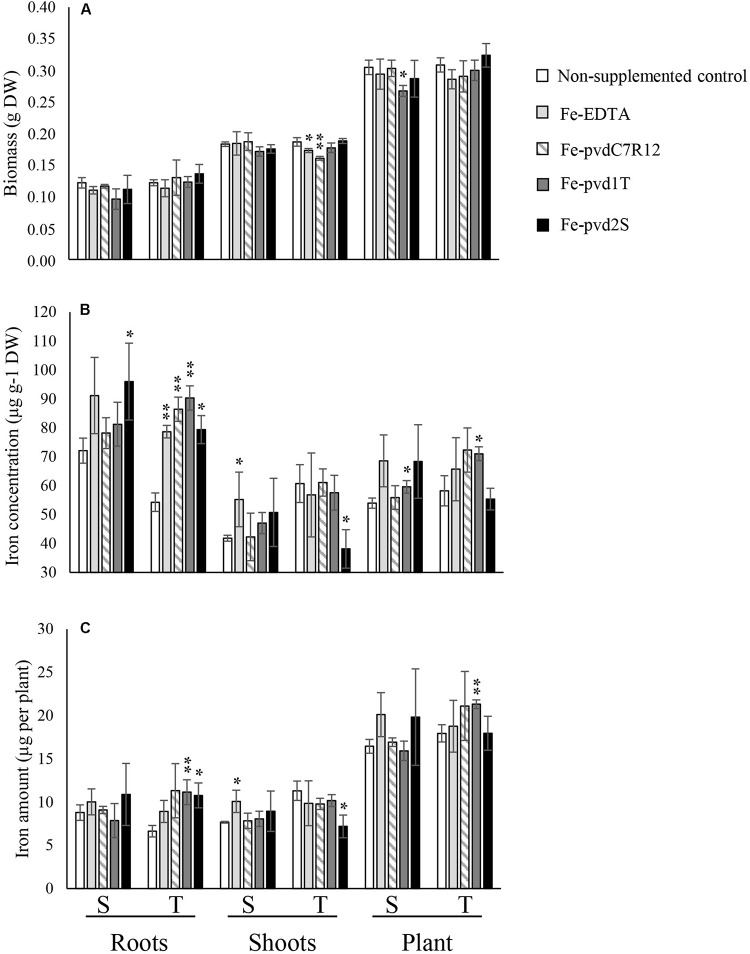
Effect of iron supplementation in the form of three ferripyoverdines, Fe-pvdC7R12, Fe-pvd1T, and Fe-pvd2S on **(A)** biomass, **(B)** iron concentration, and **(C)** iron content in two pea cultivars, tolerant (T) and susceptible (S) to iron chlorosis (*n* = 3). Error bars represent the s.d. of the mean. *P* value was calculated using one-way ANOVA between the supplementation treatment and the non-supplemented control within a same plant compartment and in a same pea cultivar. *p < 0.05; **p < 0.01.

Neither Fe-pvds nor Fe-EDTA had a significant impact on plant growth, except for plant biomass that decreased in the S cultivar in the presence of Fe-pvd1T and shoot biomass that decreased in T in the presence of Fe-pvdC7R12 and Fe-EDTA (Figure 2A).

S and T [Fe] differed significantly in the absence of supplements (Figure 2B). [Fe] was significantly higher in S (72.12 ± 4.29 μg g^–1^ DW) than in T roots (54.31 ± 4.79 μg g^–1^ DW) (*F* = 11.237, *p* = 0.028), but higher in T shoots (60.76 ± 9.81 μg g^–1^ DW) than in S shoots (41.85 ± 1.04 μg g^–1^ DW) (*F* = 16.968, *p* = 0.015), resulting in a significantly higher [Fe] R:S ratio in S (1.72 ± 0.12) than in T (0.91 ± 0.21) (*F* = 28.752, *p* = 0.006). The [Fe] R:S ratios are presented in Supplementary Table S2. Similar trends were recorded for total iron content (Supplementary Table S3).

The impact of supplementation on plant [Fe] differed significantly depending on the pvds (Figure 2). Supply of Fe-pvd1T, from a T rhizosphere strain, significantly increased the [Fe] in T roots compared to the non-supplemented controls, and this increase was also significantly higher than in roots of plants supplied with Fe-EDTA (*F* = 8.492, *p* = 0.043). Other Fe-pvds (Fe-pvdC7R12 and Fe-pvd2S) also significantly increased T [Fe] roots compared to the non-supplemented control roots.

Fe-pvd1T was the only Fe-pvd that increased plant total iron content in T compared to the non-supplemented control plants (T, 21.31 ± 0.51 μg per plant and non-supplemented control 17.94 ± 0.99 μg per plant; *F* = 27.532, *p* = 0.006) (Figure 2C). Fe-pvd1T also significantly increased [Fe] in S and T whole plants (Figure 2B). Fe-pvd2S had a negative effect on T shoot [Fe] (Figure 2B) but increased that of S roots. None of the Fe-pvds impacted [Fe] in S shoots which was only significantly increased by Fe-EDTA (Figure 2B).

Impact of Fe-pvd supplementation on plant [Fe] differed significantly depending on the pea cultivar (Table 1). Promotion of [Fe] by Fe-pvd2S, derived from a strain isolated in the S rhizosphere, was significantly greater in S than in T, whereas Fe-pvd1T from a strain isolated in the T rhizosphere, and Fe-pvdC7R12 increased [Fe] to a significantly greater extent in T plants, especially their roots. In the T cultivar, Fe-pvds, but not Fe-EDTA, significantly increased the [Fe] R:S ratio compared to the non-supplemented control (Supplementary Table S2). In the S cultivar, this [Fe] R:S ratio was not significantly modified by any of the Fe-pvds or by Fe-EDTA (Supplementary Table S2).

**TABLE 1:** Compared effect of Fe-EDTA or Fe-pyoverdine (Fe-pvdC7R12, Fe-pvd1T, and Fe-pvd2S) iron supplementation on the increased percentage iron concentration in two pea cultivars, tolerant (T) and susceptible (S) to iron chlorosis.

**Pea cultivar**	**Fe-EDTA**			**Fe-pvdC7R12**			**Fe-pvd1T**			**Fe-pvd2S**		
	**Roots**	**Shoots**	**Whole plant**	**Roots**	**Shoots**	**Whole plant**	**Roots**	**Shoots**	**Whole plant**	**Roots**	**Shoots**	**Whole plant**
S	26.4^†^ (±18.31)	32.01 (±22.59)	27.12 (±16.6)	8.43 (±7.37)	7.92 (±13.71)	4.43 (±6.64)	12.66 (±10.54)	12.48 (±8.74)	10.44 (±4)	33.09 (±18.41)	22.48 (±26.61)	26.66 (±23.56)
T	44.9 (±5.88)	8.03 (±13.91)	15.33 (±14.8)	59.13 (±10.59)	4.25 (±5.58)	24.20 (±13.11)	66.35 (±11.31)	3.21 (±5.56)	22.01 (±4.09)	46.19 (±12.31)	0	0
ANOVA/*KW p*-value	0.165	0.160	0.410	0.004**	0.961	0.080^∙^	0.005**	0.168	0.024*	0.355	*0.157*	0.026*
F/*H*	2.871	2.955	0.843	32.687	0.002	5.428	29.378	2.814	12.292	1.091	*3.019*	*11.790*

^†^Mean percentage (±SD), ^∙^*p* < 0.1; *p < 0.05; **p < 0.01. When ANOVA assumptions were not met, a Kruskall-Wallis (*KW*) test was performed; *KW p*-value and *H* are indicated in italics.

**FIGURE 3:**
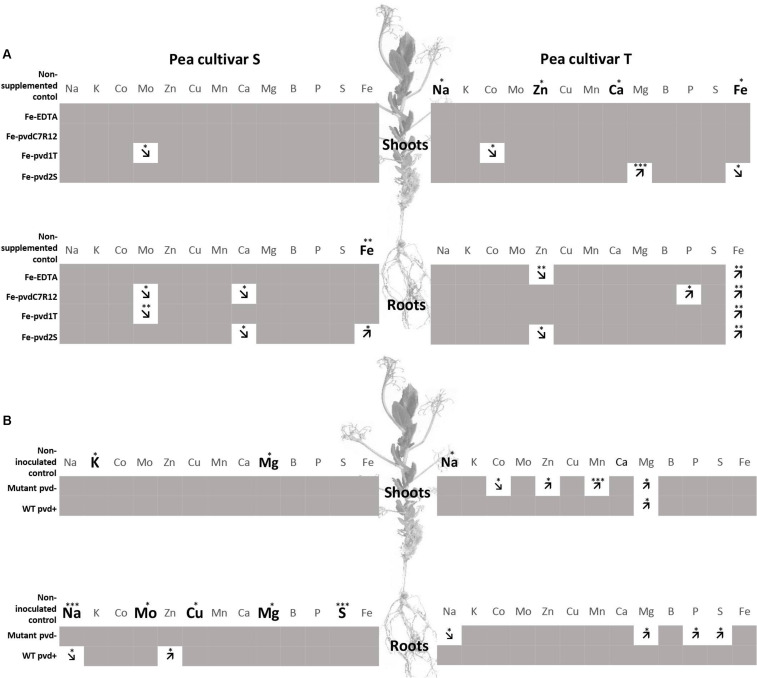
Comparison of element concentrations in two pea cultivars, tolerant (T) and susceptible (S) to iron chlorosis, grown *in vitro* (*n* = 3), **(A)** supplemented or not with Fe-EDTA, Fe-pvdC7R12, Fe-pvd1T or Fe-pvd2S, or **(B)** supplemented with Fe-EDTA (1 μM) and inoculated or not with the wild type strain of *P. fluorescens* C7R12 (WT pvd+) and its PL1 pvd− mutant (Mutant pvd−). Bold characters highlight those elements for which the concentrations were significantly higher in one cultivar, as compared to the other, within a same plant compartment, in the non-supplemented plants for panel **(A)**, and in the non-inoculated plants for panel **(B)**. Arrows indicate increase or decrease of element concentration in comparison to the corresponding control within a same plant compartment and in a same cultivar after iron supplementation for panel **(A)**, or bacterial inoculation for panel **(B)**. ANOVA *p*-values. *p < 0.05; **p < 0.01; ***p < 0.001.

When non-supplemented, the S and T cultivars also differed in their contents of other ions. As demonstrated in the field experiments, the ionomes varied according to the Fe-pvds and cultivars. Shoot [Na] was significantly higher in the T cultivar (indicated by bold letters in Figure 3A). Zinc ([Zn]) and [Ca] concentrations were also higher in T than in S shoot. Fe-pvdC7R12 had a negative effect on molybdenum ([Mo]) and [Ca] concentrations in S roots and a positive effect on [P] in T roots (indicated by arrows in Figure 3A). Fe-pvd1T induced a decrease of [Mo] in S shoots and roots and of cobalt concentration ([Co]) in T shoots. Finally, Fe-pvd2S increased [Mg] in T shoots and decreased [Ca] in S roots and [Zn] in T roots.

### Impact of *Pseudomonas* C7R12 and Its Pyoverdine-Minus Mutant on Pea Growth, Development, Iron Content and Ionome in S and T Cultivars

**FIGURE 4:**
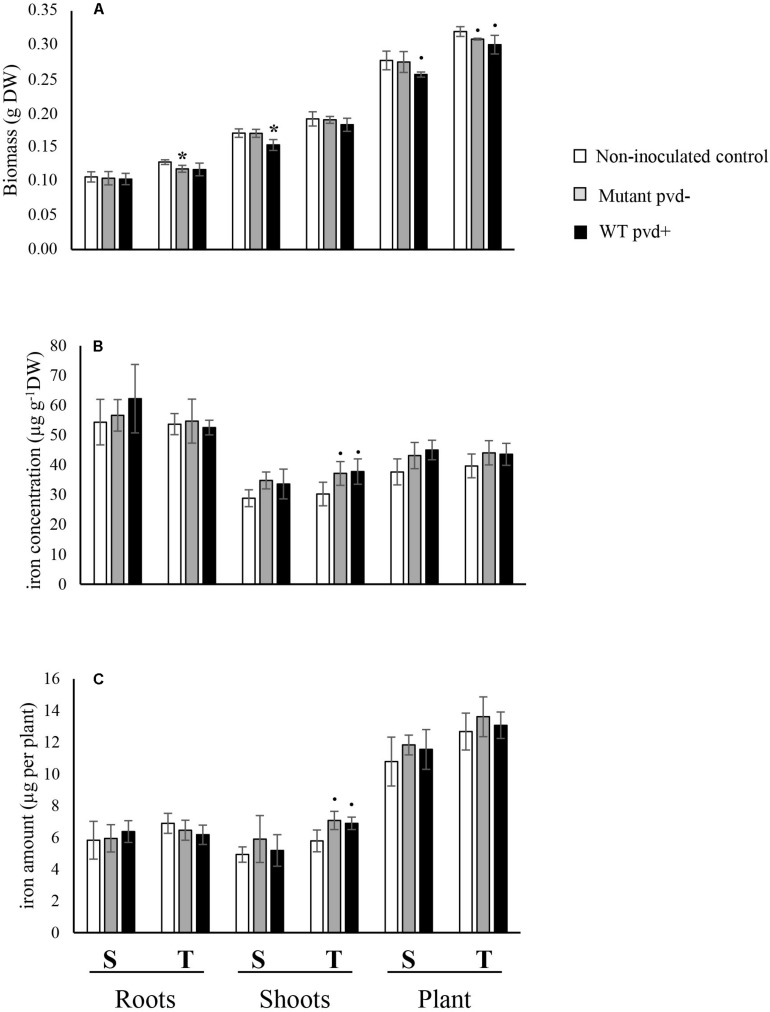
Effect of inoculation of the wild type strain *P. fluorescens* C7R12 (WT pvd+) and of PL1 its pvd− mutant (Mutant pvd−) on **(A)** biomass, **(B)** iron concentration, and **(C)** iron content in two pea cultivars, tolerant (T) and susceptible (S) to iron chlorosis (*n* = 3). Error bars represent the s.d. of the mean. *P* value was calculated using one-way ANOVA between the inoculation treatment and the non-inoculated control within a same plant compartment and in a same pea cultivar. ^∙^*p* < 0.1; *p < 0.05.

No significant interactions between the effects of cultivar and bacterial inoculation on plant growth were observed (roots *F* = 0.521, *p* = 0.607; shoot *F* = 0.706, *p* = 0.513; whole plant *F* = 0.415, *p* = 0.670) (Figure 4). Plant biomass was significantly higher in T than in S for all compartments (roots *F* = 21.238, *p* < 0.001; shoots *F* = 40.423, *p* < 0.0001) and bacterial inoculation negatively impacted the growth of shoots in both cultivars (*F* = 5.125, *p* = 0.025) and in the whole plants (*F* = 5.511, *p* = 0.02), but not in roots (*F* = 1.483, *p* = 0.266). Inoculation of C7R12 significantly decreased S shoot biomass (*F* = 8.656, *p* = 0.042) whereas its pvd− mutant significantly decreased that of root biomass in T (*F* = 7.758, *p* = 0.049) (Figure 4) but had no significant effect on S shoot biomass.

The iron concentrations in roots and shoots of non-inoculated cultivars (Figure 4B) did not differ significantly (roots, *F* = 0.018, *p* = 0.899; shoots, *F* = 0.240, *p* = 0.650).

The iron concentrations of inoculated cultivars did not differ either, except in T shoots which showed a substantially increased trend toward significance with both the WT strain and its pvd− mutant (WT pvd+, *F* = 5.081, *p* = 0.081; mutant pvd−, *F* = 7.14, *p* = 0.056) (Figure 4B). This bacterial effect on [Fe] in T shoots but not in roots led to a significant decrease of the [Fe] R:S ratio compared to that of the non-inoculated control (Supplementary Table S2).

The ionomes differed for non-inoculated peas, shoot [Na] being significantly higher in T than in S (Figure 3B). The impact of bacterial inoculation on pea ionomes depended on the cultivars and bacterial strains. In the S cultivar, the WT pvd+ strain decreased [Na] but increased [Zn] in roots. In the T cultivar, [Mg] was increased in shoots by WT and in both roots and shoots by the pvd− mutant. In this cultivar, the pvd− mutant increased [Co], [Zn], [Mn], and [Mg] in shoots and also induced a decrease of [Na] in the roots (Figure 3B). Bacterial inoculation did not impact the S shoot ionome.

## Discussion

Promoting grain legume cultivation is expected to decrease the use of chemical inputs, especially nitrogen fertilizer, diversify the protein sources for human and animal consumption, and thereby contribute to an improved sustainability of food systems (Wezel et al., 2014; Foyer et al., 2016; Zander et al., 2016; Rehman et al., 2019). However, these developments require circumvention of the bottlenecks currently associated with legume cultivation, which include an optimization of iron nutrition, to permit sufficiently high yields and increase the seed iron content, this latter being especially important in the context of partial replacement of animal proteins by plant proteins. Thus, the aim of this study was to better understand the contribution of fluorescent pseudomonads and their pyoverdines to pea adaptation to iron deficiency. For that purpose, two cultivars differing in their tolerance of chlorosis (IDC) were compared for their growth, iron content and ionome in general, when cultivated in the open field and under iron stress conditions (calcareous soil), and in bioassays with iron supplements (Fe-EDTA, Fe-pvds) and bacterial inoculation (WT pvd+ or pvd− mutant).

In the open field experiment under iron stress conditions, chlorosis was recorded in the S cultivar but not in T. This chlorosis was associated with an increased iron content in S roots compared to T roots although the total iron content did not differ between T and S plants. This is in agreement with previous reports indicating differences in iron distribution but not in total iron content between susceptible and tolerant plants (Jelali et al., 2010; Santos et al., 2015). Nor was any difference observed in the shoot and seed biomass despite a slight difference in proteins composition. Collectively, this field experiment confirmed the difference in susceptibility to iron stress of the two cultivars S and T although their crop yield and seed quality (i.e., iron content) was comparable.

*In vitro*, in the absence of iron supplementation, no difference in whole-plant iron concentration was observed, but the distribution of iron within the plant differed between the two cultivars, the [Fe] R:S ratio being significantly higher in S than in T. This observation on young plants *in vitro* further confirmed the results obtained on mature plants in the field at harvest time which indicated differences in iron distribution between IDC tolerant and susceptible plants.

The contribution of pyoverdines to differential adaptation of the two pea cultivars to iron stress was further explored during *in vitro* bioassays. The ferripyoverdine Fe-pvd1T, as compared with Fe-EDTA, promoted root iron concentration in the tolerant cultivar (T). This confirms and extends to a new plant species and other pyoverdines results previously reported (Vansuyt et al., 2007; Jin et al., 2010; Shirley et al., 2011). In the tolerant cultivar T, the impact of Fe-pvd1T and Fe-pvd2S on concentration and on total iron content in the shoots and whole plant was significantly different. In contrast, neither Fe-pvds differently impacted the concentration not the total amount of iron in the susceptible cultivar S. These results support our hypotheses that (i) distinct pvds could differently impact plant iron nutrition and (ii) that distinct plant genotypes could be differently impacted by pvds. They further suggest that differences between the S and T cultivar in valuing iron chelated to pyoverdines could possibly account for their different susceptibility to iron stress; this hypothesis is currently being tested. Comparison of the increased percentage of iron concentration induced by Fe supplements in the two pea cultivars showed that Fe-pvd1T was more favorable to iron nutrition in T whereas Fe-pvd2S had a more significant impact on S. This suggests that the two pvds produced by strains isolated from peas had a most favorable impact on the cultivar from which their producing strain originated. In the absence of iron supplementation, no difference was observed in the global whole-plant iron concentration but the distribution of iron within the plant differed between the two pea cultivars, the [Fe] R:S ratio being significantly higher in S than in T. This observation on young plants *in vitro* and on mature plants at harvest time in the field, is in agreement with previous reports of distinguishing traits between IDC tolerant and susceptible plants. The iron content of chlorotic peas was shown to be as high as in non-chlorotic peas indicating that iron homeostasis and distribution *in planta*, rather than iron acquisition, would be involved in tolerance to IDC (Barhoumi et al., 2007; Jelali et al., 2010; Yakop, 2012). This was also evidenced in soybean (Bloom et al., 2011; Santos et al., 2015). Our results also demonstrated that iron distribution *in planta* could be significantly impacted by *Pseudomonas* pyoverdines siderophores. Our results also revealed another distinction between the tolerant cultivar, T, and the susceptible cultivar, S. Indeed, after Fe-pvds supplementation a significant modification of iron distribution, as evaluated by the [Fe] R:S ratio, was only recorded in the tolerant cultivar, T.

Inoculation assays with the WT pvd+ strain C7R12 and its pvd− mutant PL1 were performed to study the effects of the model pyoverdine pvdC7R12 produced *in situ* by the bacterium. Bacterial inoculation with WT pvd+ and the mutant pvd− led to slight increases in shoot iron concentration. However, a significant modification of the [Fe] R:S ratio was observed, which again concerned the tolerant T cultivar, but not the susceptible S cultivar. This effect could not be ascribed to pvdC7R12 since WT pvd+ and the pvd− mutant had a similar impact on plant iron status. Nevertheless, it shows that the strain C7R12 could modify pea iron status in T and confirms the greater sensitivity of iron distribution to microbial activity in the tolerant than in the susceptible cultivar. However, Fe-pvds and microbial strains differently modified iron distribution in T. Several factors may account for this discrepancy. Since iron was present in limited amounts under *in vitro* conditions, a small amount of Fe-EDTA was added in order to avoid competition for iron between plant and bacteria. Although low, this Fe-EDTA supplementation of the agar medium modified the experimental conditions in such a way as to affect the iron status of the pea plants. Indeed, the differences observed between iron concentrations in roots and shoots of the S and T non-supplemented control plants were no longer detectable between S and T non-inoculated control plants. In addition to modifying pea iron status, these experimental conditions could also have hampered the synthesis and/or effect of the pvd. This could explain the lack of difference between the effect of the WT pvd+ and the mutant pvd− on pea iron content. Moreover, besides pyoverdine, fluorescent pseudomonads synthesize secondary siderophores and many other compounds reported to affect plant development (Haas and Défago, 2005; Cornelis, 2010). In particular, in addition to secondary siderophores, strain C7R12 produces indole acetic acid and harbors a type III secretion system that may account for the effects of inoculation (Viollet et al., 2017).

Our results also demonstrated that Fe-pvd supplementation modified the plant concentrations of ions other than iron. This is in agreement with previous reports that the plant ionome is closely linked to the plant’s nutritional status (Cohen et al., 1998; Maillard et al., 2016). As observed for iron, the reported modifications varied with the pvds and pea cultivars. This shows that microbial activity impacts elements, besides iron, that are essential for plant growth and development (e.g., P, S, Mn) and for the nutritional value of staple foods (e.g., Zn, Mg).

Interestingly, a higher concentration of shoot Na in T than in S was consistently observed in the field and *in vitro*, which was not altered by iron supplementation or bacterial inoculation. In *Arabidopsis thaliana*, the *AtHKT*1;1 locus, encoding a sodium transporter, has been identified as a major factor controlling natural variation in leaf Na^+^ accumulation and a weak allele of *AtHKT*1;1, that drives elevated leaf Na^+^, has been linked to elevated salinity tolerance (Rus et al., 2006; Baxter et al., 2010). Since a correlation between greater tolerance to saline-alkaline stress and high efficiency of iron acquisition has been observed in rice (Li et al., 2016), further analysis of leaf Na accumulation in relation to salinity and IDC tolerance in various peas cv. could be of interest.

Collectively, our results show for the first time that pyoverdines of fluorescent pseudomonads may impact positively iron nutrition in peas and, on an even broader scale, their elemental nutrition. They further stress the specificity of the corresponding interactions between pea cultivars and pseudomonads. Indeed, the iron nutrition of T cultivar benefited more than that of S from bacterial siderophores. Also, allocation of iron within the plant was modified by the bacteria and ferrisiderophores in T but not in S cultivar. These differences indicate that fluorescent pseudomonads and their pyoverdines impact more iron nutrition and allocation in T than in S; this would suggest that pseudomonads and pyoverdines could possibly account for cultivar differences in IDC tolerance. This hypothesis calls for taking better account the specificity of interactions between plant genotype and associated microorganisms in future attempts to promote plant iron nutrition. Experiments are undergoing to test these hypothesis and proposal.

## Data Availability Statement

All datasets generated for this study are included in the article/Supplementary Material.

## Author Contributions

SM and PL initiated the study. TL, PL, and SM conceived and designed the experiments, analyzed the data, and wrote the manuscript. TL, CC, and CB performed the experiments. CJ, CS, and PD provided expertise in pea genotypes and field experiments. KG contributed to the analyses and interpretations of seed proteins. AO and FL performed the plant ionome. All authors contributed to the discussion and approved the final manuscript.

## Conflict of Interest

The authors declare that the research was conducted in the absence of any commercial or financial relationships that could be construed as a potential conflict of interest.
