# Supplementary material for: Impact of Bacterial Siderophores on Iron Status and Ionome in Pea
Source: Front Plant Sci. 2020 Jun 12;11:730. doi: 10.3389/fpls.2020.00730 (PMC7304161; doi:10.3389/fpls.2020.00730)
Supplement: Supplementary file 1 [file Data_Sheet_1.docx]

**
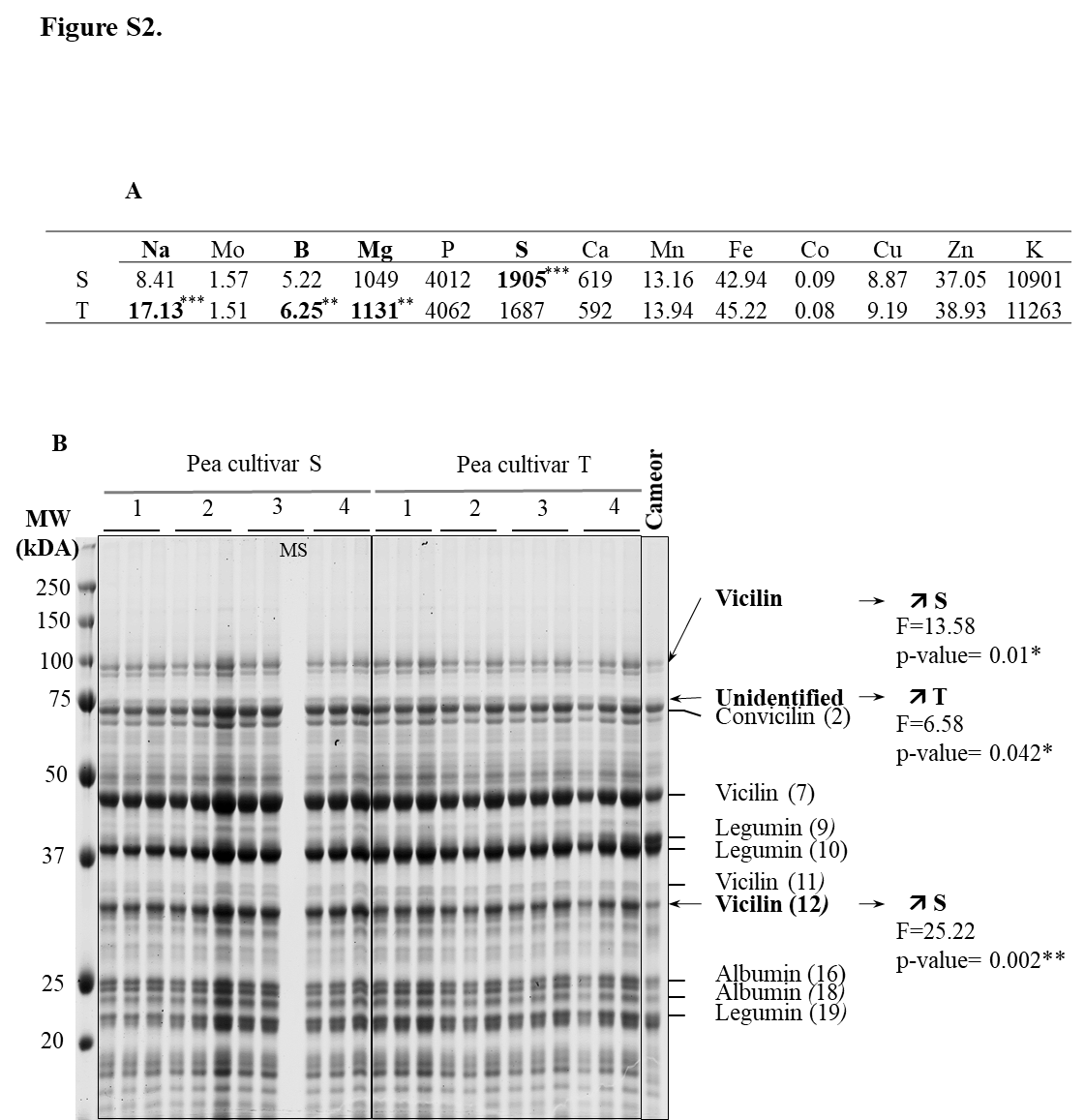
**

**Figure S1.** Comparison of mature seeds from a susceptible, S, and a tolerant, T, pea cultivar grown in a calcareous loam soil under field conditions. (A), Element compositions of mature seeds of pea cv. S and pea cv. T, mean value (N=3) in mg kg^-1^. Bold characters highlight those elements for which the concentrations in the seeds were significantly higher in one cultivar as compared to the other. One-way ANOVA p-values: *, p<0.05; **, p<0.01; ***, p<0.001. (B), Comparison of protein compositions of mature seeds from both pea cultivars. Pea cv. Cameor was used as a reference for protein band annotation. The numbers in brackets refer to protein band identification as defined by Henriet *et al.*, (2019). Bold names highlight proteins found in higher amounts in one cv. (indicated to the right of an up-pointing arrow together with corresponding ANOVA F and p-values) than in the other. MS, Missing sample.
