# Supplementary material for: Impact of Bacterial Siderophores on Iron Status and Ionome in Pea
Source: Front Plant Sci. 2020 Jun 12;11:730. doi: 10.3389/fpls.2020.00730 (PMC7304161; doi:10.3389/fpls.2020.00730)
Supplement: Supplementary file 2 [file Table_1.docx]

**Table S1.** Physicochemical characteristics of the Montardoise soil in which two pea cultivars, tolerant (T) and susceptible (S) to iron chlorosis, were grown under field conditions. Four bulk soil samples were analyzed independently. Soil analyses were performed commercially by the INRA Laboratory of Soil Analysis, Arras, France (Fulfils the requirements of the standard NF EN ISO/IEC 17025).

|  | **Soil characteristics** | |  | **Mean (n=4)** | ***± SD**** |
| --- | --- | --- | --- | --- | --- |
|  | pH |  | | **8.27** | *0.083* |
| Granulometry | clays (< 2µm) | g/kg | | **287.25** | *12.447* |
|  | fine silts (2/20 µm) | g/kg | | **297.50** | *10.472* |
|  | coarse silts (50/200µm) | g/kg | | **139.00** | *2.708* |
|  | fine sands (50/200µm) | g/kg | | **91.00** | *0.816* |
|  | coarse sands (200/2000µm) | g/kg | | **185.25** | *11.087* |
| Miscellaneous | organic carbon | g/kg | | **19.45** | *1.511* |
|  | total nitrogen | g/kg | | **2.29** | *0.150* |
|  | total CaO_3_ | g/kg | | **641.25** | *22.292* |
|  | organic matter | g/kg | | **33.68** | *2.613* |
|  | C/N |  | | **8.50** | *0.332* |
|  | Phosphorus (P_2_O_5_) (Joret-Hebert) | g/kg | | **0.23** | *0.026* |
|  | Phosphorus (P_2_O_5_) (Olsen) | g/kg | | **0.07** | *0.009* |
|  | CEC Metson | cmol/kg | | **11.13** | *0.499* |
|  | CEC cobaltihexamine | cmol/kg | | **15.68** | *0.922* |
| Cobaltihexamine Exchangeable  (ICP-AES/EAF) | Calcium | cmol/kg | | **16.88** | *0.690* |
|  | Magnesium | cmol/kg | | **0.59** | *0.028* |
|  | Sodium | cmol/kg | | **0.03** | *0.002* |
|  | Potassium | cmol/kg | | **0.96** | *0.103* |
|  | Iron | cmol/kg | | **nd** | nd |
|  | Manganese | cmol/kg | | **nd** | nd |
|  | Aluminium | cmol/kg | | **nd** | nd |
| EDTA Extractable  (ICP-AES) | Copper | mg/kg | | **2.09** | *0.086* |
|  | Iron | mg/kg | | **4.97** | *0.215* |
|  | Manganese | mg/kg | | **9.82** | *2.009* |
|  | Zinc | mg/kg | | **6.36** | *0.679* |
| DTPA Extractable  (ICP-AES) | Copper | mg/kg | | **1.30** | *0.074* |
|  | Iron | mg/kg | | **10.32** | *0.362* |
|  | Manganese | mg/kg | | **7.67** | *1.236* |
|  | Zinc | mg/kg | | **3.98** | *0.350* |
| Tamm method  (ICP-AES) | Silicium | g/100g | | **0.03** | *0.003* |
|  | Aluminium | g/100g | | **0.16** | *0.013* |
|  | Iron | g/100g | | **0.08** | *0.006* |
| Merha-Jackson method (ICP-AES) | Silicium | g/100g | | **0.06** | *0.004* |
|  | Aluminium | g/100g | | **0.11** | *0.005* |
|  | Iron | g/100g | | **0.53** | *0.028* |
| Total elements  (HF - ICP-AES) | Aluminium | g/100g | | **2.10** | *0.129* |
|  | Calcium | g/100g | | **25.33** | *1.069* |
|  | Iron | g/100g | | **1.02** | *0.064* |
|  | Potassium | g/100g | | **0.54** | *0.030* |
|  | Magnesium | g/100g | | **0.25** | *0.009* |
|  | Manganese | mg/100g | | **568.50** | *16.381* |
|  | Sodium | g/100g | | **0.12** | *0.006* |
|  | Phosphorus | g/100g | | **0.29** | *0.010* |
|  | Sulfur | mg/kg | | **479.25** | *17.385* |

*. SD: standard deviation; ICP-AES; Inductively coupled argon plasma - atomic emission spectroscopy; EAF. Electric arc furnace; HF. complete solubilization in hydrofluoric acid.
