# Supplementary material for: Impact of Bacterial Siderophores on Iron Status and Ionome in Pea
Source: Front Plant Sci. 2020 Jun 12;11:730. doi: 10.3389/fpls.2020.00730 (PMC7304161; doi:10.3389/fpls.2020.00730)
Supplement: Supplementary file 3 [file Table_2.docx]

**Table S2.** Root:shoot ratios of iron concentration, [Fe] R:S, observed in two pea cultivars, tolerant (T) and susceptible (S) to iron chlorosis, grown *in vitro* and (**A**) supplemented or not with Fe-EDTA, Fe-pvdC7R12, Fe-pvd1T or Fe-pvd2S, or (**B**) supplemented with Fe-EDTA (1µM) and inoculated or not with the wild type strain of *P. fluorescens* C7R12 (WT pvd+) and its PL1 pvd- mutant (Mutant pvd-). One-way ANOVA F-value and p-value were calculated between the supplementation treatment and the corresponding control (non-supplemented or non-inoculated). Bold characters highlight significant differences. *, p<0.05.

**A**

| Pea cultivar | Treatments | [Fe] R:S | ANOVA^†^  F-value | p-value |
| --- | --- | --- | --- | --- |
| S | Non-supplemented | 1.72 ±0.12 |  |  |
|  | Fe-EDTA | 1.67 ±0.27 | 0.10 | 0.76 |
|  | Fe-pvdC7R12 | 1.90 ±0.43 | 0.41 | 0.56 |
|  | Fe-pvd1T | 1.74 ±0.31 | 2.54 | 0.19 |
|  | Fe-pvd2S | 1.93 ±0.37 | 0.88 | 0.40 |
| T | Non-supplemented | 0.91 ±0.21 |  |  |
|  | Fe-EDTA | 1.55 ±0.70 | 2.54 | 0.19 |
|  | Fe-pvdC7R12 | 1.42 ±0.06 | **13.90** | **0.02*** |
|  | Fe-pvd1T | 1.60 ±0.31 | **10.36** | **0.03*** |
|  | Fe-pvd2S | 2.21 ±0.77 | **9.83** | **0.04*** |

**B**

| Pea cultivar | Treatments | [Fe] R:S | ANOVA^†^  F-value | p-value |
| --- | --- | --- | --- | --- |
| S | Non-inoculated | 1.88 ±0.21 |  |  |
|  | WT pvd+ | 1.87 ±0.37 | 0.01 | 0.94 |
|  | Mutant pvd- | 1.72 ±0.51 | 0.30 | 0.61 |
| T | Non-inoculated | 1.79 ±0.13 |  |  |
|  | WT pvd+ | 1.40 ±0.01 | **18.24** | **0.01*** |
|  | Mutant pvd- | 1.47 ±0.12 | **9.17** | **0.04*** |

^†^, [Fe] R:S ratio was subjected to arsine transformation prior to one-way ANOVA.
