# Supplementary material for: Impact of Bacterial Siderophores on Iron Status and Ionome in Pea
Source: Front Plant Sci. 2020 Jun 12;11:730. doi: 10.3389/fpls.2020.00730 (PMC7304161; doi:10.3389/fpls.2020.00730)
Supplement: Supplementary file 4 [file Table_3.docx]

**Table S3.** Comparison of iron concentration and iron content between two pea cultivars tolerant (T) and susceptible (S) to iron chlorosis grown without iron supplementation (Non-supplemented control).

| Pea cultivars | Iron concentration (µg g^-1^ DW) | | | Iron content (µg per plant DW) | | |
| --- | --- | --- | --- | --- | --- | --- |
|  | Roots | Shoots | Whole plant | Roots | Shoots | Whole plant |
| S | 72.12 ±4.29^†^ | 41.85 ±1.04 | 53.96 ±1.78 | 8.79 ±0.89 | 7.66 ±0.10 | 16.45 ±0.80 |
| T | 54.31 ±4.79 | 60.76 ±9.81 | 58.72 ±5.23 | 6.63 ±0.67 | 11.31 ±1.53 | 17.94 ±0.99 |
| ANOVA |  |  |  |  |  |  |
| F-value | 22.381 | 12.680 | 1.814 | 11.257 | 16.960 | 4.120 |
| p-value | **0.009**** | **0.023*** | 0.249 | **0.028*** | **0.014*** | 0.112 |

^†^, Mean ±SD; *****, p<0.05; ******, p<0.01.
